
JOURNAL INFORMATION
==============================
NLM Title Abbreviation: BJPsych Open
Journal ID: BJPsych Open
NLM Journal ID: 101667931
Journal ID: BJO

BJPsych Open

EISSN: 2056-4724
Publisher: Royal College of Psychiatrists

ARTICLE INFORMATION
==============================
PMCID: PMC13329318
DOI: 10.1192/bjo.2026.12030
Article ID: S2056472426120304
Article version: 1
Subjects: Front Cover (OFC, IFC) and matter

BJO volume 12 issue S1 Cover and Front matter

Publication date: 2026 Jun
Electronic publication date: 2026 Jun 30
Volume: 12
Issue: Suppl 1
Issue title: Abstracts from the RCPsych International Congress 2026, 15–18 June
First page: f1
Last page: f5
Copyright: © Royal College of Psychiatrists 2026
Copyright year: 2026
Copyright holder: Royal College of Psychiatrists
License: This is an Open Access article, distributed under the terms of the Creative Commons Attribution licence (http://creativecommons.org/licenses/by/4.0/), which permits unrestricted re-use, distribution, and reproduction in any medium, provided the original work is properly cited.
License URL: https://creativecommons.org/licenses/by/4.0/

Page count: 5
displayText: Supplement
tdm-reservation: 1
tdm-policy: https://www.cambridge.org/tdm/tdmrep-policy.json

==============================

JUNE 2026 | VOL12 NO S1

RCPSYCH ROYAL COLLEGE OF PSYCHIATRISTS

### BJPsych open

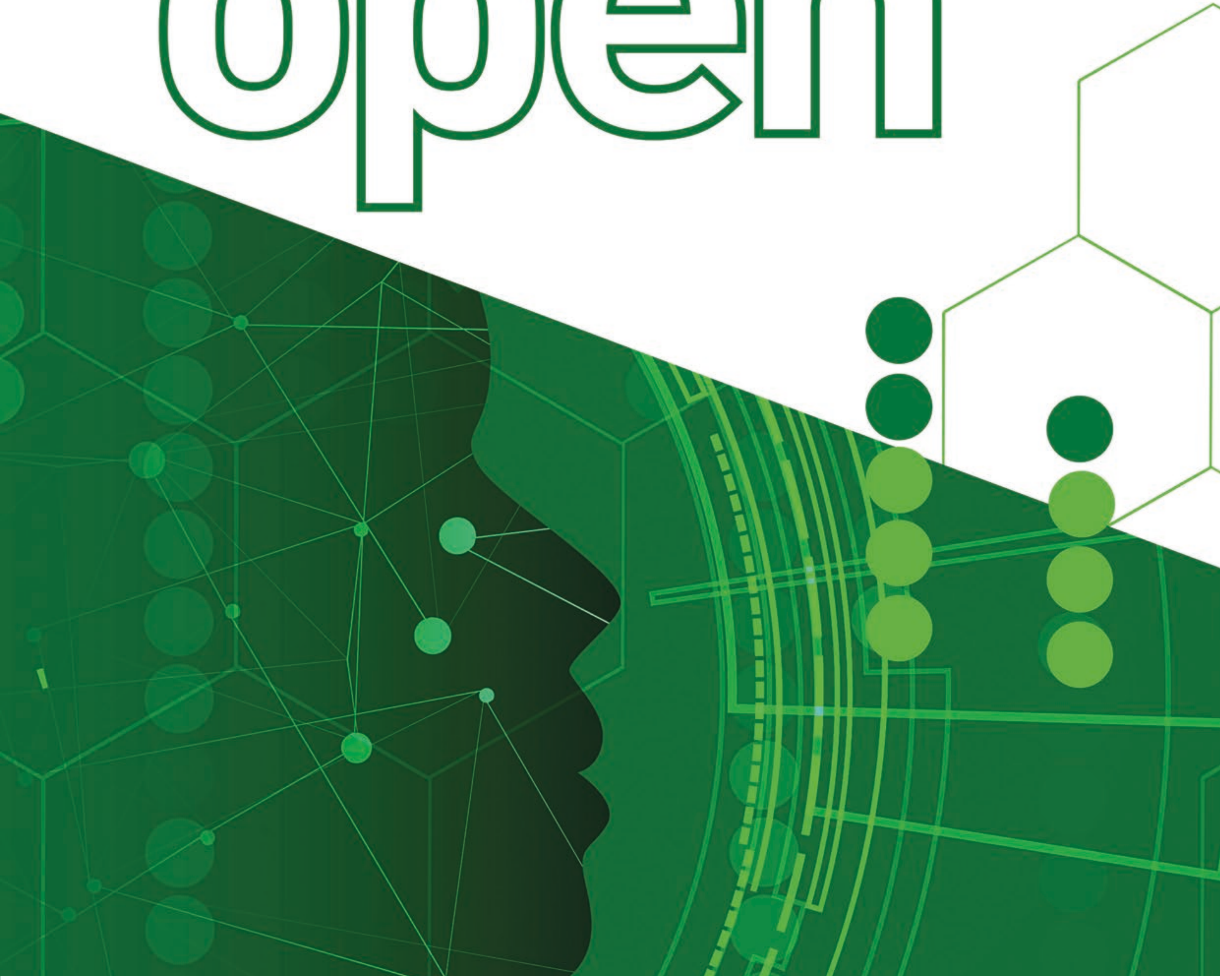

### RCPSYCH INTERNATIONAL CONGRESS 2026 ABSTRACTS

ROYAL COLLEGE OF PSYCHIATRISTS
International Congress

### ABSTRACTS

The submitted abstracts of posters presented at the RCPsych International Congress 2026, 15–18 June, are published here in a special supplement to *BJPsych Open*.

**Peer review process**:
Abstract review was managed by the RCPsych Academic Faculty rather than by the standard *BJPsych Open* peer review process and should not be quoted as peer reviewed by *BJPsych Open* in any subsequent publication.

**Disclaimer**:
The abstracts have been published using author-supplied copy, with only minor editing made to correct spelling, style, and format where appropriate. No responsibility is assumed for any claims, instruction or methods contained in the abstracts and it is recommended that these be verified independently.

Unless so stated, material in this supplement does not necessarily reflect the views of the Royal College of Psychiatrists, RCPsych Academic Faculty or Editor-in-Chief. The publishers are not responsible for any error of omission or fact.

The abstracts are Open Access, distributed under the terms of the Creative Commons Attribution licence (https://creativecommons.org/licenses/by/4.0/), which permits unrestricted re-use, distribution, and reproduction in any medium, provided the original work is properly cited.

### Congress Organising Committee:

### Congress Executive Committee

Professor Fiona Gaughran (Appointed role)
Professor Sridevi Sira Mahalingappa (Congress co-chair)
Dr Abdul Raoof (Associate Dean for Advanced Learning and Conferences)
Professor Rohit Shankar (Congress co-chair)
Professor Allan Young (Chair of the RCPsych Academic Faculty)

### Congress Advisory Board

Professor Alka Ahuja
Dr Salim Al-Huseini
Professor Mohammed Al-Uzri
Dr Julie Anderson

Dr Nish Babu Mathew
Professor Owen Bowden-Jones
Dr Calin Cavaropol
Professor Sam Chamberlain
Ms Elen Cook
Dr Sanjukta Das
Professor Subodh Dave
Dr Jana de Villiers
Dr Dina Elgabry
Mr Mark Farmer
Professor Saeed Farooq
Professor Naomi Fineberg
Dr Jonathan Gibb
Dr Rachel Gibbons
Dr Sundar Gnanavel
Dr Mike Groves
Dr Sumeet Gupta
Dr Ian Hall
Dr Claire-Marie Hosein
Dr Declan Hyland
Professor Kenneth Kaufman
Dr Helen Killaspy
Professor Ania Korszun
Dr Fergus Lewis
Dr Pallab Majumder
Ms Em McAllister
Dr Omer Moghraby
Dr Jane Morris
Dr Jo O'Reilly
Dr Saju Padakkara
Dr Lokapriya Premalatha
Dr Thomas Rourke
Dr Amrit Sachar
Dr Lade Smith
Dr Toral Thomas
Dr Alex Thomson
Dr Alex Till
Dr Ovais Wadoo

Volume 12 – Supplement 1 – June 2026

*BJPsych Open* (ISSN 2056-4724 (Online) 2026 (volume 12). Published by Cambridge University Press.
Contents of the Supplement are available on the journal website: https://www.cambridge.org/core/journals/bjpsych-open

### CONTENTS

### Abstracts from the RCPsych International Congress 2026, 15–18 June

| | |
|---|---|
| Peer review process/disclaimer | S1 |
| Congress organising committee | S1 |
| Rapid-Fire Presentations | S1–S16 |
| Accepted Poster | |
| Research | S16–S91 |
| Education and Training | S92–S130 |
| Quality Improvement | S130–S219 |
| Service Evaluation | S220–S269 |
| Audit | S269–S355 |
| Case Study | S355–S395 |
| Psychopharmacology | S395–S405 |
